# Dietary Nutrition and Gut Microbiota Composition in Patients With Hypertensive Disorders of Pregnancy

**DOI:** 10.3389/fnut.2022.862892

**Published:** 2022-04-06

**Authors:** Jinran Yu, Bo Zhang, Tingting Miao, Haiting Hu, Yongye Sun

**Affiliations:** ^1^Department of Nutrition and Food Hygiene, School of Public Health, Qingdao University, Qingdao, China; ^2^Department of Child Healthcare, Shanghai Center for Women and Children's Health, Shanghai, China; ^3^Department of Education, Changzhou Maternity and Child Health Care Hospital Affiliated to Nanjing Medical University, Changzhou, China

**Keywords:** hypertensive disorders of pregnancy, blood pressure, dietary nutrition, gut microbiota, third trimester

## Abstract

**Objective:**

The aim is to explore the intakes of dietary nutrients and the changes of gut microbiota composition among patients with hypertensive disorders of pregnancy (HDP) and provide a theoretical basis for the prevention and treatment of HDP.

**Methods:**

This study was conducted at the Maternal and Child Health Care Hospital of Changzhou. A total of 170 pregnant women (72 patients with HDP in the case group and 98 healthy pregnant women in the control group) in the third trimester were enrolled. Dietary nutrient intakes were assessed through a food frequency questionnaire survey. Fresh fecal samples were aseptically collected, and 16S rDNA sequencing was conducted. The intakes of dietary nutrients and the diversity and relative abundance of gut microbiota were compared between pregnant women with and without HDP. A logistic regression model was used to investigate the association between differential gut microbial genera and the risk of HDP.

**Results:**

The daily dietary intakes of vitamin A and vitamin C in pregnant women with HDP were significantly lower than those in the control group. The relative abundances of Bacteroidota, Bacteroidaceae, and *Bacteroides* were increased, and the relative abundances of Actinobacteriota, Lachnospiraceae, Prevotellaceae, Bifidobacteriaceae, *Blautia, Prevotella*, and *Bifidobacterium* were decreased in women with HDP compared with those in the controls. In addition, the relative abundance of *Bifidobacterium* was positively correlated with dietary intakes of vitamin C and vitamin E in patients with HDP. After adjustment for confounding factors, the odds ratio (95% confidence interval) of HDP for the relative abundance of *Bifidobacterium* was 0.899 (0.813, 0.995).

**Conclusion:**

The composition of gut microbiota in pregnant women with HDP was significantly changed compared with that of healthy controls. The relative abundance of *Bifidobacterium* was negatively associated with HDP. Moreover, dietary vitamin C and gut *Bifidobacterium* may cooperatively contribute to reduce the risk of HDP.

## Introduction

Hypertensive disorders of pregnancy (HDP) is mainly divided into gestational hypertension (GH), preeclampsia (PE), chronic hypertension complicated with PE, and pregnancy complicated with chronic hypertension (1). A meta-analysis reported that the overall prevalence of HDP was 7.6% in China (2). The World Health Organization found that ~10.7% of deaths was caused by HDP among 2,443,000 maternal deaths in 115 countries (3). HDP is a risk factor for hypertension, diabetes, Alzheimer's disease, and ischemic heart disease (4–6).

The pathogenesis of HDP has not been fully understood, but it is believed to be caused by the combined maternal, placental, and fetal effects. The occurrence and development of HDP are accompanied by a series of pathophysiological changes, such as immune maladjustment, chronic uterine placental ischemia (7), oxidative stress (8), abnormal angiogenic factors (9), and placental fat infiltration (10). Nutrition plays an important role in the aforementioned changes (8). Grum et al. demonstrated that women who received nutritional counseling during pregnancy had a significantly reduced risk of PE compared to those who did not receive counseling (11). A case–control study showed that daily supplementation of vitamin C and vitamin E reduced the risk of PE (12).

Dietary nutrition is closely related to the composition of gut microbiota, which may influence the homeostasis and biological processes of humans directly and also through the metabolites produced by microbial fermentation of nutrients, especially short-chain fatty acids (SCFAs) (13). Lutein, an antioxidant carotenoid, was shown to facilitate the growth of bifidobacteria and lactobacilli, and reduce the growth of *Bacteroides spp*. and *Clostridium spp*. (14). On the other hand, Karlsson et al. found a high level of serum beta-carotene in healthy controls, which was probably produced by gut microbiota (15). These studies suggest that gut microbiota in turn plays an important role in mediating the protective effects of dietary nutrition in humans. In pregnant women with future HDP, a higher intake of dietary fiber elevated the abundance of *Veillonella*, which had anti-inflammatory properties (16). In recent years, some studies have explored the differences in gut microbiota between pregnant women with and without HDP. A study in Shanghai found that compared with pregnant women with PE, Bifidobacteriaceae was enriched in the control group (17). However, Altemani et al. drew an opposite conclusion among obese patients with PE in Australia (18). Liu et al. found that the levels of *Clostridium perfringens* and *Bulleidia moorei* were significantly increased, and *Coprococcus catus* was decreased among women with PE in Guangzhou (19), which may reduce blood pressure by regulating the metabolism of propionic acid to promote the formation of SCFAs.

The aforementioned studies of gut microbiota in patients with HDP showed inconsistent results. In addition, multiple confounding factors, such as race, region, diet, and lifestyle, limit the extrapolation of these results. To date, very few studies have considered nutrient intakes in the study of gut microbiota differences between women with and without HDP. Here, we hypothesize that dietary nutrition and gut microbiota cooperatively contribute to HDP. We explored the intakes of dietary nutrients and the changes in gut microbiota composition among patients with HDP in the third trimester in Changzhou. We also analyzed the correlations of blood pressure and gut microbiota with clinical characteristics and dietary nutrients. The current work would provide a theoretical basis for the prevention and treatment of HDP.

## Methods

### Study Population

Pregnant women with HDP in the third trimester who planned to deliver in the Maternal and Child Health Care Hospital of Changzhou from October 2019 to October 2020 were selected as the case group, and healthy pregnant women hospitalized during the same period (±1 week) were selected as the control group. The inclusion criteria for patients with HDP were based on “Hypertensive disorders of pregnancy: ISSHP classification, diagnosis & management recommendations for international practice” (1), including GH, PE, chronic hypertension complicated with PE, and pregnancy complicated with chronic hypertension. All subjects were ≥20 years old, ≥28 gestational weeks, and had good compliance and communication skills. The exclusion criteria for subjects were as follows: (1) smoking and/or drinking during pregnancy; (2) used antibiotics and/or taken probiotics during the past month; (3) suffering from inflammatory bowel disease, infectious disease, heart disease, liver or kidney disease, immune system disease, mental disease, or malignancy.

### Sample Size

Based on Yusuf's study of dietary vitamin C intake (20), *P*_0_ = 27.9%, *OR* = 4.1. According to the following formula, the sample size of both the case group and the control group was calculated to be 42 each:


$$\begin{array}{l} n=\frac{{\left[Z_{1-\frac{\alpha }{2}}\sqrt{2\bar{P}\left(1-\bar{P}\right)}+Z_{\beta }\sqrt{P_{1}\left(1-P_{1}\right)+P_{0}\left(1-P_{0}\right)}\right]}^{2}}{{\left(P_{1}-P_{0}\right)}^{2}} \end{array}$$

Among them, $P_{1}=\frac{\left(OR\times P_{0}\right)}{\left(1-P_{0}+OR\times P_{0}\right)}$, $\bar{P}=\frac{\left(P_{1}+P_{0}\right)}{2}$, α = 0.05, β = 0.1. Finally, a total of 170 pregnant women were enrolled in this study, including 72 in the case group and 98 in the control group.

### Laboratory Examination

Fasting blood samples were collected in coagulation tubes to detect blood biochemical indexes, including total cholesterol (TC), triglyceride (TG), high-density lipoprotein-cholesterol (HDL-C), low-density lipoprotein-cholesterol (LDL-C), total bilirubin, albumin, alanine aminotransferase (ALT), aspartate aminotransferase (AST), uric acid, and C-reactive protein (CRP), by an automatic biochemical analyzer (AU5800).

Serum vitamin levels, including vitamin A, vitamin E, and beta-carotene, were detected by high-performance liquid chromatography (Agilent 1260). The standard was dissolved in anhydrous ethanol to prepare the standard reserve solution. A total amount of 200 μl of serum samples stored in dark was added to anhydrous ethanol to remove proteins and then extracted with n-hexane. After centrifugation, the supernatant was blow-dried with nitrogen at ambient temperature, redissolved in methanol, and detected after filtration. Finally, the standard curve was drawn to calculate the content of vitamins.

### Dietary Assessment

Dietary intake during the previous 4 weeks was assessed using a food frequency questionnaire (FFQ), which was conducted face-to-face by trained dietitians. The questionnaire was modified on the basis of the questionnaire used in the Chinese nutrition and health surveillance. Participants were asked to report the consumption frequency and the average consumption of food items per time according to food pictures labeled with standard portion sizes. The daily average consumption of each item was calculated by the consumption frequency and the average consumption per time. Finally, the data were imported and computed to obtain the nutrient intakes by using NCCW 12.0 (Qingdao University, China), which is primarily based on China Food Composition 2002 and 2004.

### 16S Amplicon Sequencing

At least 500 mg of fresh feces from each participant was aseptically collected, then transferred and stored at a −80°C freezer within 2 h. Fecal bacterial genomic DNA was extracted using MagPure Stool DNA KF kit B (Magen, China), and 16S rDNA V4-region sequencing was conducted based on Illumina HiSeq 2500 by Huada Medical Laboratory Co., Ltd. (Wuhan, China). DNA samples were amplified with the primers 515F (5'-GTGCCAGCMGCCGCGGTAA-3') and 806R (5'-GGACTACHVGGGTWTCTAAT-3'). The pairs of reads obtained by paired-end sequencing were joined to obtain Tags by using FLASH 1.2 (21). The Tags were clustered with 97% similarity to generate operational taxonomic unit (OTU) by UPARSE 7.0 (22). Then compared Tags with OTU representative sequence were used to obtain the OTU abundance table by using the USEARCH_global method (23). The information of annotation was obtained by comparing the table of OTU abundance with the SILVA 138 database (update 2020.08) (24).

### Statistical Analysis

Student's *t*-test or Mann–Whitney *U*-test was used to compare the mean levels of continuous variables with normal distributions or with non-normal distributions between pregnant women with and without HDP. Chi-square test was used to compare the distribution of categorical variables between groups by using SPSS 23.0. The alpha-diversity (including indexes of Shannon, Simpson, Chao1, and Observed species) and relative abundance of gut microbiota were estimated online (https://www.microbiomeanalyst.ca./), and GraphPad Prism 8.0 and ggplot2 package in R 4.0.3 were used for visualization. The beta-diversity of gut microbiota was estimated using R package vegan based on Bray-Curtis distance, principal coordinate analysis (PCoA) was plotted with R package ggplot2 for visualization, and permutational MANOVA was used for comparison among groups. LEfSe analysis was performed online (https://huttenhower.sph.harvard.edu/galaxy/) based on linear discriminant analysis (LDA). The default value of LDA was 2. Correlation analysis was performed based on Spearman's ranking, and heat map was used for visualization by HemI 1.0. A logistic regression model was used to estimate odds ratios (*OR*s) with 95% confidence intervals (*CI*s) of the association between differential gut microbial genera and HDP risk. Model 1 was a crude model, and model 2 was adjusted for covariates as follows: age, gestational week, pre-pregnancy body mass index (BMI), and parity (assignment: 0 = 0, 1 = 1 or over). Moreover, bootstrap method was used to evaluate the stability of regression model. All *p* values were two-sided and *p* < 0.05 was considered significant.

## Results

### Clinical Characteristics

The clinical characteristics of the case group and the control group are summarized in Table 1. The pre-pregnancy BMI, systolic blood pressure (SBP), diastolic blood pressure (DBP), and proportion of primiparas in patients with HDP were significantly higher than those in the controls, and the gestational week in patients with HDP was significantly lower than that in the controls (*p* < 0.05). Subjects with HDP had significantly elevated serum levels of AST, uric acid, and CRP, and significantly lower serum levels of total bilirubin, albumin, and vitamin A than those without HDP (*p* < 0.05).

**Table 1:** Clinical characteristics of the two groups.

	**Case (*n* = 72)**	**Control (*n* = 98)**	**T/Z/ χ^2^**	***P***
Age (years)	29.50 ± 4.98	29.63 ± 3.97	0.193	0.847
Gestational week (weeks)	37.86 (35.57, 38.86)	38.57 (37.25, 39.18)	−2.538	0.011
Pre-pregnancy BMI (kg/m^2^)	23.95 (21.66, 27.55)	21.45 (19.00, 24.13)	−4.335	<0.001
Maternal weight gain (kg)	15.70 (12.75, 20.00)	15.00 (11.50, 16.45)	−1.937	0.053
SBP (mmHg)	140.50 (135.00, 148.75)	117.00 (110.00, 128.25)	−9.522	<0.001
DBP (mmHg)	90.00 (81.25, 94.00)	70.50 (67.00, 76.00)	−9.599	<0.001
Parity (*n*, %)			4.581	0.032
0	45.00 (62.50%)	45.00 (45.90%)		
≥1	27.00 (37.50%)	53.00 (54.10%)		
Occupation (*n*, %)			1.135	0.287
Yes	38.00 (66.70%)	60.00 (75.00%)		
No	19.00 (33.30%)	20.00 (25.00%)		
TC (mmol/L)	6.02 (4.95, 7.04)	5.93 (5.30, 6.48)	−0.181	0.856
TG (mmol/L)	3.75 (2.73, 5.56)	3.66 (2.81, 4.84)	−0.322	0.747
HDL-C (mmol/L)	2.06 (1.83, 2.29)	1.95 (1.72, 2.23)	−1.073	0.283
LDL-C (mmol/L)	3.47 ± 0.98	3.38 ± 0.68	−0.620	0.536
Total bilirubin (μmol/L)	5.90 (4.90, 7.40)	7.20 (6.25, 9.10)	−4.439	<0.001
Albumin (g/L)	35.40 ± 3.89	37.24 ± 2.54	3.687	<0.001
ALT (U/L)	10.00 (8.00, 14.70)	9.40 (7.93, 12.33)	−1.332	0.183
AST (U/L)	17.70 (14.68, 22.95)	16.55 (14.73, 19.85)	−1.983	0.047
Uric acid (μmol/L)	355.80 (285.13, 457.03)	290.75 (253.70, 335.43)	−5.038	<0.001
CRP (mg/L)	5.40 (3.40, 9.60)	3.70 (2.20, 6.50)	−2.017	0.044
Vitamin A (μg/mL)	0.41 ± 0.20	0.50 ± 0.21	2.013	0.047
Vitamin E (μg/mL)	9.58 (6.71, 11.93)	9.78 (8.22, 13.36)	−1.312	0.189
Beta-carotene (μg/mL)	0.11 (0.05, 0.33)	0.19 (0.12, 0.31)	−0.788	0.431

*Data are presented as mean ± SD for continuous variables with normal distributions, median (P_25_, P_75_) for continuous variables with non-normal distributions, or participants (percentage) for categorical variables. BMI, body mass index; SBP, systolic blood pressure; DBP, diastolic blood pressure; TC, total cholesterol; TG, triglyceride; HDL-C, high-density lipoprotein-cholesterol; LDL-C, low-density lipoprotein-cholesterol; ALT, alanine aminotransferase; AST, aspartate aminotransferase; CRP, C-reactive protein*.

### Dietary Nutrient Intakes

A total of 91 FFQs were collected. As shown in Table 2, the daily dietary intakes of vitamin A and vitamin C in pregnant women with HDP were significantly lower than those in the control group (*p* < 0.05). No significant differences in other dietary nutrient intakes were detected between the two groups (*p* > 0.05).

**Table 2:** Comparison of dietary nutrient intakes per day between the two groups.

	**Case (*n* = 44)**	**Control (*n* = 47)**	**T/Z**	***P***
Energy (kcal)	1,840.80 ± 550.59	1,982.89 ± 640.34	1.330	0.186
The ratio of energy supplied by protein (%)	17.13 ± 3.78	16.94 ± 4.25	−0.272	0.786
The ratio of energy supplied by fat (%)	36.86 ± 7.66	36.37 ± 7.75	−0.353	0.725
The ratio of energy supplied by carbohydrate (%)	46.24 ± 9.02	47.05 ± 9.53	0.487	0.627
Fiber (g)	11.49 (8.53, 18.14)	13.43 (9.61, 18.02)	−1.013	0.311
Cholesterol (mg)	179.79 (113.97, 287.31)	199.15 (113.56, 279.43)	−0.127	0.899
Vitamin A (μg)	666.76 (525.39, 901.18)	909.07 (642.70, 1,162.31)	−2.390	0.017
Vitamin C (mg)	106.04 (79.64, 185.69)	159.92 (114.84, 225.35)	−2.113	0.035
Vitamin E (mg)	27.77 (24.38, 34.52)	28.65 (23.28, 34.86)	−0.369	0.712
Potassium (mg)	2,166.72 ± 773.52	2,275.33 ± 980.50	0.584	0.561
Sodium (mg)	635.76 (509.36, 766.94)	615.66 (505.25, 1,003.52)	−0.373	0.709
Calcium (mg)	664.63 (435.10, 863.84)	601.31 (456.21, 728.91)	−0.421	0.674
Magnesium (mg)	303.19 (245.35, 358.53)	312.00 (235.43, 389.30)	−0.095	0.924
Iron (mg)	20.54 (16.13, 26.24)	20.89 (16.14, 26.26)	−0.107	0.915
Zinc (mg)	11.26 ± 3.86	11.07 ± 4.66	−0.216	0.829
Selenium (mg)	45.58 (33.68, 62.44)	47.81 (32.50, 62.34)	−0.012	0.990

*Data are presented as mean ± SD for continuous variables with normal distributions or median (P_25_, P_75_) for continuous variables with non-normal distributions*.

### Correlation Analysis of Blood Pressure

**Figure 1:**
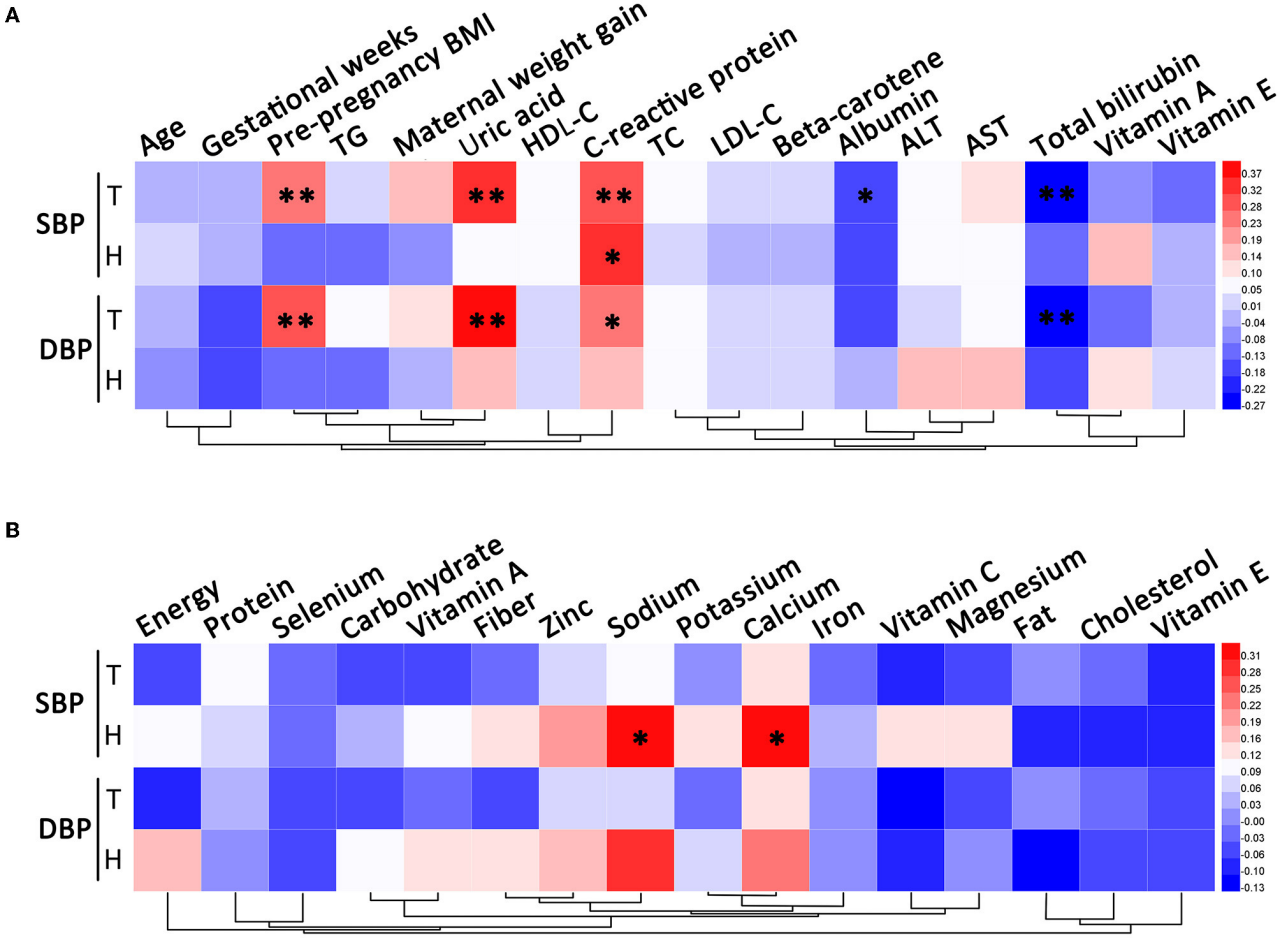
Correlation analysis of blood pressure. **(A)** The correlations between blood pressure and clinical characteristics. **(B)** The correlations between blood pressure and dietary nutrient intakes. T, total; H, HDP. *P < 0.05, **P < 0.01.

Systolic blood pressure and DBP were both positively correlated with pre-pregnancy BMI, uric acid, and CRP, and negatively correlated with total bilirubin in all subjects. For all participants, albumin was negatively correlated with SBP. Among patients with HDP, SBP was positively correlated with CRP and dietary intakes of sodium and calcium (Figure 1).

### Diversity of Gut Microbiota

**Figure 2:**
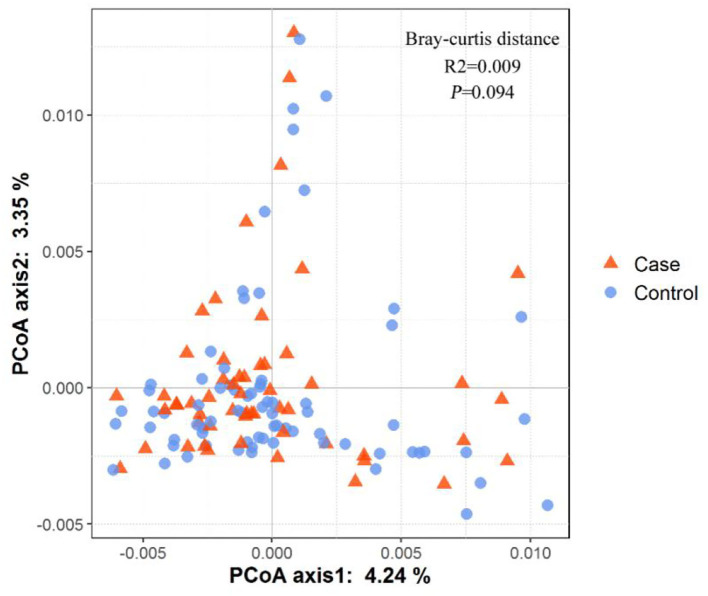
The beta-diversity of gut microbiota between the two groups.

**Figure 3:**
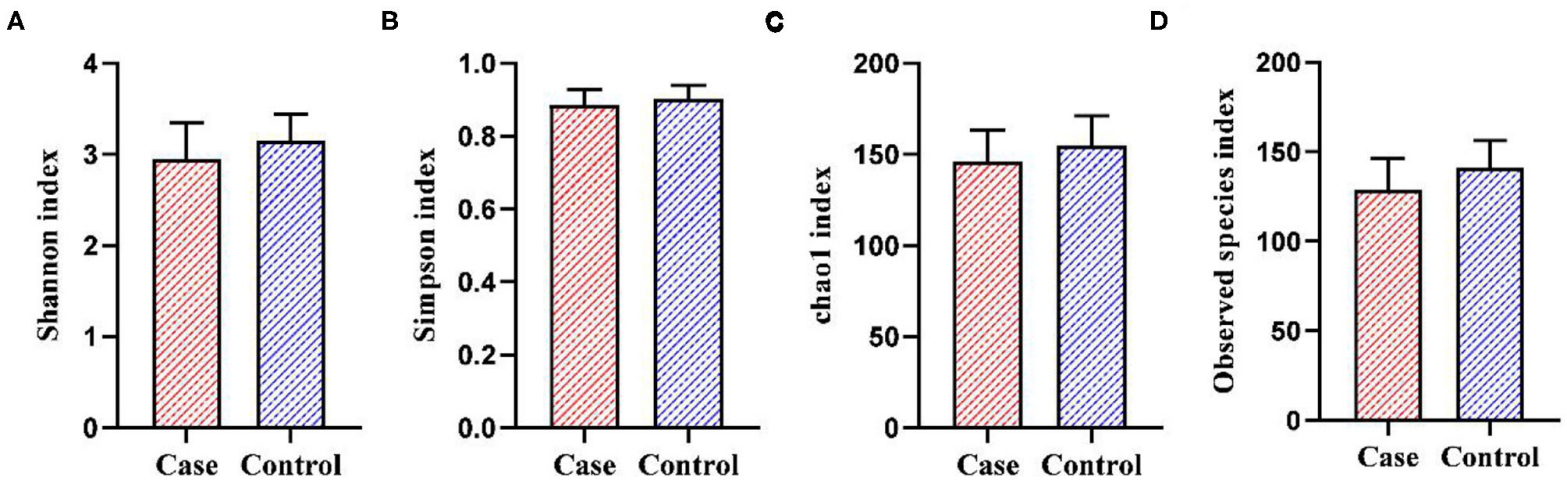
The alpha-diversity of gut microbiota between the two groups. **(A)** Shannon index. **(B)** Simpson index. **(C)** Chao1 index. **(D)** Observed species index.

A total of 129 fecal samples were collected (55 from the case group and 74 from the control group). A total of 5,306,609 reads were obtained by 16S rDNA sequencing and clustered into 4,622 OTUs. As shown in Figure 2, PCoA plot showed that the beta-diversity between the two groups did not differ significantly (R^2^ = 0.009, *p* = 0.094). As shown in Figure 3, the Shannon index (*p* = 0.076) and Chao1 index (*p* = 0.058) of gut microbiota in the case group had a decreasing trend compared with those in the control group, though the difference was not statistically significant. The Simpson index (*p* = 0.126) and Observed species index (*p* = 0.101) were not significantly different between the two groups.

### Relative Abundance of Gut Microbiota

**Figure 4:**
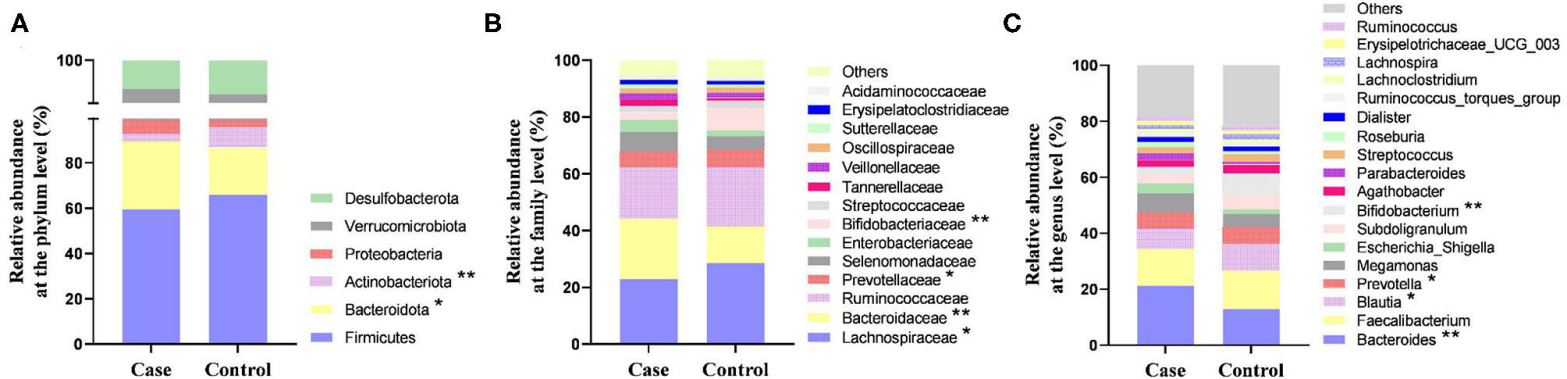
The relative abundance of gut microbiota between the two groups. **(A)** Relative abundance at the phylum level. **(B)** Relative abundance at the family level. **(C)** Relative abundance at the genus level. *P < 0.05, **P < 0.01.

At the phylum level, the gut microbiota of the two groups consisted of mainly Firmicutes, Bacteroidota, Actinobacteriota, and Proteobacteria. The relative abundance of Bacteroidota in women with HDP was significantly higher than that in the control group, and the relative abundance of Actinobacteriota was significantly lower than that in the control group (*p* < 0.05). The relative abundances of Firmicutes and Proteobacteria showed no significant difference between the two groups (*p* > 0.05) (Figure 4A).

**Figure 5:**
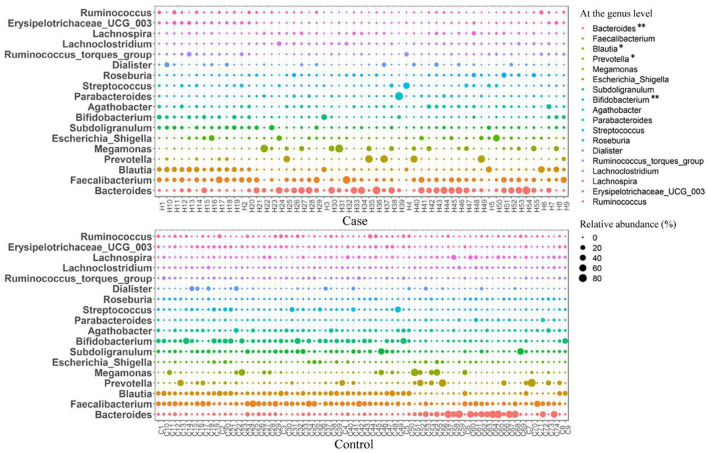
Bubble diagram of the relative abundance of gut microbiota at the genus level between the two groups. *p < 0.05, **p < 0.01.

At the family level, the relative abundance of Bacteroidaceae in the case group was significantly higher than that in the control group, and the relative abundances of Lachnospiraceae, Prevotellaceae, and Bifidobacteriaceae were significantly lower than those in the control group (*p* < 0.05) (Figure 4B). At the genus level, the relative abundance of *Bacteroides* in women with HDP was significantly higher than that in the control group, and the relative abundances of *Blautia, Prevotella*, and *Bifidobacterium* were significantly lower than those in the control group (*p* < 0.05) (Figures 4C, 5).

**Figure 6:**
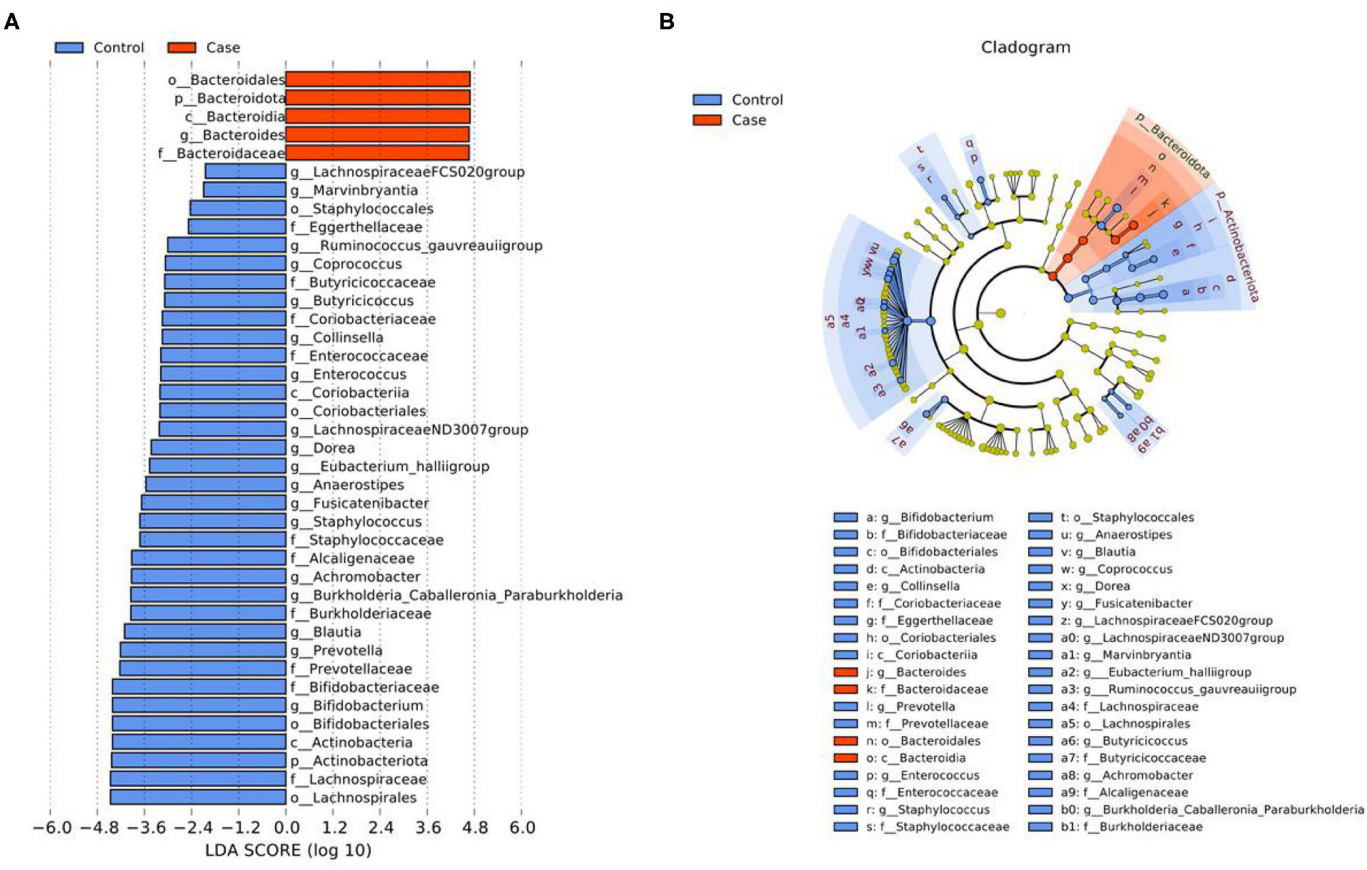
LEfSe analysis of gut microbiota between the two groups. **(A)** LDA value distribution diagram. **(B)** Cladogram. p, Phylum; c, Class; o, Order; f, Family; g, Genus.

The differential microbiota and their contribution at five levels from phylum to genus between the case group and the control group were identified by LEfSe analysis (Figure 6). A total of forty differential microbiota were detected between the two groups, all of which had a log LDA score >2. Five and thirty-five microbiota were enriched in the case group and the control group, respectively.

### Correlation Analysis of Differential Gut Microbial Genera

**Figure 7:**
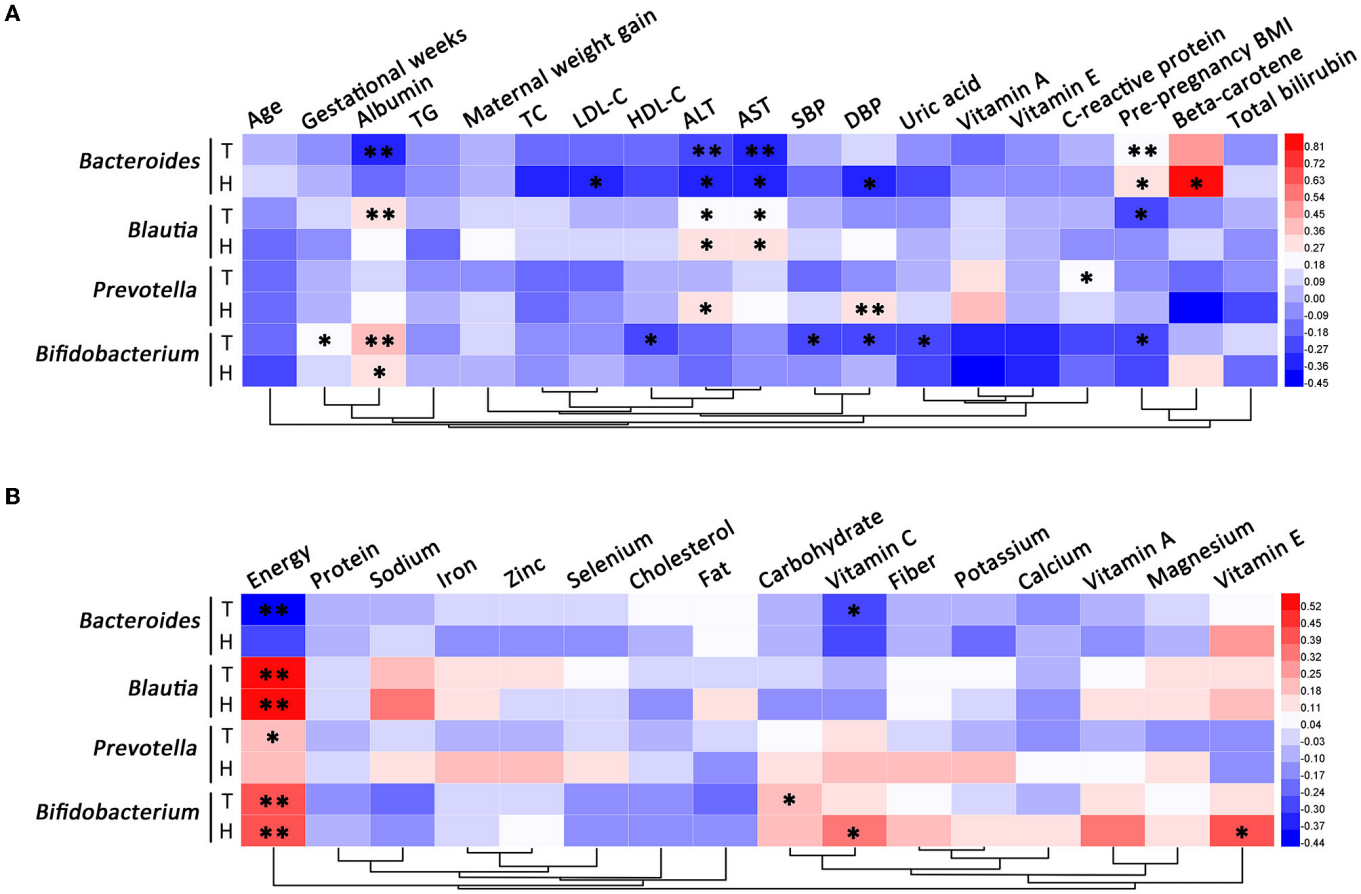
Correlation analysis of differential gut microbial genera. **(A)** The correlations between differential gut microbial genera and clinical characteristics. **(B)** The correlations between differential gut microbial genera and dietary nutrient intakes. T, total; H, HDP. *P < 0.05, **P < 0.01.

For pregnant women with HDP, the relative abundance of *Bacteroides* was positively correlated with pre-pregnancy BMI and serum beta-carotene, and negatively correlated with LDL-C, ALT, AST, and DBP. The relative abundance of *Blautia* was positively correlated with ALT, AST, and dietary energy intake in the case group. Among subjects with HDP, the relative abundance of *Prevotella* was positively correlated with ALT and DBP. The relative abundance of *Bifidobacterium* was positively correlated with albumin and dietary intakes of energy, vitamin C, and vitamin E in patients with HDP (Figure 7).

### Logistic Regression Analysis of Differential Gut Microbial Genera and HDP Risk

In the unadjusted model (model 1), the *OR*s (95% *CI*s) of HDP indicated that the relative abundance of *Bacteroides* was positively related to HDP (1.020 [1.002, 1.038]), and the relative abundance of *Bifidobacterium* was negatively associated with HDP (0.898 [0.837, 0.963]). After adjustment for age, gestational week, pre-pregnancy BMI, and parity (model 2), the relative abundance of *Bifidobacterium* remained significantly negatively associated with HDP (0.899 [0.813, 0.995]). The results of Bootstrap indicated a good stability of these associations (*p* < 0.05). However, the association between the relative abundance of *Bacteroides* and HDP was no longer statistically significant in model 2 (Table 3).

**Table 3:** *OR*s with 95% *CI*s for the association between differential gut microbial genera and the risk of HDP.

	**Model 1**		**Model 2**	
	**β (95%*CI*)**	***OR*** **(95%*CI*)**	**β (95%*CI*)**	***OR*** **(95%*CI*)**
*Bacteroides*	0.020 (0.002, 0.043)^*^	1.020 (1.002, 1.038)^*^	0.007 (−0.017, 0.039)	1.007 (0.985, 1.029)
*Blautia*	−0.023 (−0.074, 0.015)	0.977 (0.940, 1.015)	−0.031 (−0.106, 0.026)	0.970 (0.917, 1.025)
*Prevotella*	−0.002 (−0.046, 0.023)	0.998 (0.976, 1.021)	0.001 (−0.051, 0.035)	1.001 (0.973, 1.031)
*Bifidobacterium*	−0.108 (−0.233, −0.043)^*^	0.898 (0.837, 0.963)^**^	−0.106 (−0.287, −0.043)^*^	0.899 (0.813, 0.995)^*^

*Model 1, crude model. Model 2, adjusted for age, gestational week, pre-pregnancy BMI, and parity. OR, odds ratio; CI, confidence interval*.

* *P < 0.05*,

** *P < 0.01*.

## Discussion

Hypertensive disorders of pregnancy is a complication of pregnancy that severely threatens maternal and infant health, and is the second leading cause of maternal death worldwide (3). The prevention and treatment of HDP is an important public health concern. Multiple risk factors have been proposed for the development of HDP, for example, inflammatory response, endothelial dysfunction, unbalanced diet (8), and gut microbiota (19). The imbalance of gut microbiota in pregnant women may affect the remodeling of maternal physiological balance, leading to a decline in adaptability and a variety of adverse pregnancy outcomes such as PE, intrauterine growth retardation, and spontaneous abortion (25). In the present study, we found that there were significant differences in dietary nutrition as well as gut microbiota composition between late pregnancy women with HDP and without HDP. Furthermore, the relative abundance of *Bifidobacterium* was significantly negatively associated with HDP.

We found a significant reduction in dietary intakes of vitamin A and vitamin C among women with HDP compared with healthy controls. A cross-sectional study found that women with PE tended to intake lower vitamin A compared to non-PE women (26). The serum level of vitamin A in patients with HDP in this study was also significantly lower than that in the control group, which was consistent with several studies on patients with PE (27, 28). Data from Denmark (29) and Jordan (20) suggested that the reduced intake of vitamin C was associated with an increased risk of PE. The mechanisms underlying the association may be related to impaired oxidation resistance or elevated inflammation level because of oxidative stress (30). In addition, we found a decreased serum level of total bilirubin in subjects with HDP compared with the controls. Data from National Health and Nutrition Examination Survey [2009-2012] revealed that a higher serum bilirubin level reduced hypertension risk by inactivating and inhibiting the production of reactive oxygen species in vascular cells (31). Wannamethee et al. demonstrated that both the dietary vitamin C intake and plasma vitamin C level were negatively correlated with CRP, which is a marker of endothelial dysfunction (32). As expected, a higher level of CRP was observed in patients with HDP in the current study.

Our study showed that there was no significant difference in alpha-diversity between the two groups, but the Shannon index and Chao1 index in the case group had a decreasing trend compared with the control group, suggesting a lower richness and uniformity of gut microbiota in patients with HDP compared with that in the control group. Similarly, previous studies found that the alpha-diversity of pregnant women with PE in the third trimester also showed a decreasing trend compared with the controls, though the difference was not statistically significant (18, 33, 34).

In our study, Bacteroidota, Bacteroidaceae, and *Bacteroides* were enriched in pregnant women with HDP. Bacteroidota is a gram-negative bacteria that produces lipopolysaccharide (LPS) (35), and the elevated relative abundance of Bacteroidota suggest that the load of intestinal LPS in patients with HDP may also increase. In animals, LPS induced pathological conditions similar to PE (36). In addition, *Bacteroides spp*. was shown to play a harmful role in hypertension (37). Studies on patients with PE in the third trimester were consistent with the results of the current study (33, 34).

We found that the relative abundance of Actinobacteriota in pregnant women with HDP was significantly decreased compared with the controls. Actinobacteriota among patients with primary hypertension also showed a decreasing trend (37). However, two recent studies involving women with PE contradicted our result (18, 33). The role of Actinobacteriota in HDP needs to be further clarified. A significant decrease in Prevotellaceae and *Prevotella* was revealed among patients with HDP in this study. *Prevotella* may produce SCFAs using fiber and polysaccharides (38). In addition, studies in China suggested that *Prevotella* promoted the occurrence of hypertension by inflammation potentially (39, 40).

In this study, the relative abundances of Bifidobacteriaceae and *Bifidobacterium* in patients with HDP decreased compared with the healthy controls, which was consistent with the trend in a previous study of patients with PE in Shanghai (17) and patients with hypertension in Tangshan (39) and Henan (40). *Bifidobacterium* plays an important role in maintaining health (41). *Bifidobacterium* can maintain the homeostasis of gut microbiota, protect intestinal mucosal barrier, and reduce LPS (42). An animal study indicated that gut *Bifidobacterium* in rats with PE increased after supplementation of probiotics, which may control blood pressure through the activation of angiotensin-converting enzyme II (43). We found a positive correlation between *Bifidobacterium* and albumin level. A decreased albumin level indicates an increased permeability of cell membrane due to arteriospasm in women with HDP. This correlation suggested that *Bifidobacterium* and albumin may cooperatively contribute to HDP.

We reported a novel observation that the relative abundance of *Bifidobacterium* was positively correlated with dietary intake of antioxidant vitamin C in patients with HDP. In a spontaneously hypertensive rat model, the anti-hypertensive function of vitamin C supplementation via improving the diversity and abundance of gut microbiota was confirmed (44). Meanwhile, inflammatory response and oxidative stress related to up-regulated blood pressure were reduced in hypertensive rats after treatment with vitamin C. Similarly, in humans, vitamin C significantly increased microbial alpha-diversity and fecal SCFAs compared to placebo (45). However, vitamin C increased the level of *Bifidobacterium in vitro* but not in humans. Interestingly, in both *in vitro* and *in vivo* experiments, intestinal vitamin C uptake was inhibited by LPS (46), which may be reduced by *Bifidobacterium*.

The present study also found that intestinal butyrate-producing Lachnospiraceae and *Blautia* were at a low level in patients with HDP. Lachnospiraceae was decreased in women with PE (17, 34), which may be explained by inhibiting the production of plasminogen activator inhibitor-1 through butyrate to reduce blood pressure (47). Previous results on gut *Blautia* in patients with PE (17) and patients with hypertension (39) were consistent with this study. In animals, the abundance of *Blautia* was negatively correlated with SBP (48). However, Lv et al. found that *Blautia* was enriched in patients with PE (49).

To our knowledge, this is one of the first studies to explore the relationship between dietary vitamin and mineral intake and gut microbiota in patients with HDP. Second, we took the lead in using the adjusted logistic regression model to estimate the association between differential gut microbial genera and HDP risk. Our study also has some limitations. First, the causality cannot be determined because of the case–control design of the study. Large longitudinal studies are needed to investigate the associations between dietary nutrition, gut microbiota, and HDP. Second, nutrient supplementation and fecal transplantation in animal models are needed to clarify the underlying mechanisms regarding the relationships between dietary nutrient intake, gut microbiota, and HDP.

In conclusion, the gut microbiota composition of patients with HDP was significantly changed compared with healthy controls. The relative abundance of *Bacteroides* was increased, whereas the relative abundances of *Blautia, Prevotella*, and *Bifidobacterium* were decreased in patients with HDP compared with the controls. The relative abundance of *Bifidobacterium* was significantly negatively associated with HDP. Moreover, dietary vitamin C and gut *Bifidobacterium* may cooperatively contribute to reduce the risk of HDP.

## Data Availability Statement

The datasets presented in this study can be found in online repositories. The name of the repository and accession number can be found here: National Center for Biotechnology Information (NCBI) BioProject, https://www.ncbi.nlm.nih.gov/bioproject/, PRJNA800588.

## Ethics Statement

The studies involving human participants were reviewed and approved by the Ethics Committee of Changzhou Maternal and Child Health Care Hospital (QNRC2016302). The patients/participants provided their written informed consent to participate in this study.

## Author Contributions

JY and YS conceived and designed the study. JY, BZ, TM, and HH recruited clinical participants, collected samples, and conducted the questionnaire survey. JY drafted the manuscript. YS critically revised the manuscript. All authors read and approved the final manuscript.

## Funding

This study was supported by the Young Talents Science and Technology Project of Changzhou Health Commission (No. QN202050).

## Conflict of Interest

The authors declare that the research was conducted in the absence of any commercial or financial relationships that could be construed as a potential conflict of interest.

## Publisher's Note

All claims expressed in this article are solely those of the authors and do not necessarily represent those of their affiliated organizations, or those of the publisher, the editors and the reviewers. Any product that may be evaluated in this article, or claim that may be made by its manufacturer, is not guaranteed or endorsed by the publisher.
